# Cardiac tissue oxidative stress and inflammation after vitamin D administrations in high fat- diet induced obese rats

**DOI:** 10.1186/s12872-017-0597-z

**Published:** 2017-06-19

**Authors:** Mahdieh Abbasalizad Farhangi, Ghazaleh Nameni, Ghazaleh Hajiluian, Mehran Mesgari-Abbasi

**Affiliations:** 1 Drug Applied Research Center, Nutrition Research Center, Department of Community Nutrition, Tabriz University of Medical Sciences, Attar Neyshabouri Street, Tabriz, Iran; 2 Student Research Committee, Neuroscience Research Center, Tabriz University of Medical Sciences, Tabriz, Iran; 3 Nutrition Research Center, Department of Community Nutrition, Tabriz University of Medical Sciences, Tabriz, Iran

**Keywords:** Vitamin D, Cardiac tissue, Oxidative stress, Obesity, Inflammation

## Abstract

**Background:**

Obesity is associated with numerous metabolic and inflammatory disorders. The current study was aimed to evaluate the effects of vitamin D administration on the markers of oxidative stress and inflammation in the cardiac tissue of high-fat diet induced obese rats.

**Methods:**

In the beginning of the study, 40 male Wistar rats were divided into two groups: normal diet (ND) and high fat diet (HFD) for 16 weeks; then each group subdivided into two groups including: ND, ND + vitamin D, HFD and HFD + vitamin D. Vitamin D supplementation was done for 5 weeks at 500 IU/kg dosage. Tumor necrosis factor (TNF)-α concentration and markers of oxidative stress including glutathione peroxidase (GPx), superoxide dismutase (SOD), malondialdehyde (MDA) and catalase (CAT) concentrations in the cardiac tissue and serum concentrations of lipids in rats were determined using ELISA kits and spectrophotometry methods respectively.

**Results:**

According to our results, GPx activity in ND and ND + vitamin D group was significantly higher compared with HFD group. Similarly, SOD activity was also significantly increased in ND + vitamin D group compared with ND and HFD groups. Moreover, vitamin D administration, significantly reduced catalase activity in ND + vitamin D and HFD + vitamin D groups (*P* < 0.05). TNF-α concentration in heart tissue in ND + vitamin D group significantly reduced compared with ND group. Cardiac tissue MDA concentration in baseline or after vitamin D administration did not changed significantly.

**Conclusion:**

Vitamin D improved cardiac oxidative stress and inflammatory markers in HFD induced obese rats. Further studies in human models are needed to further confirm the use of this nutrient in daily clinical practice.

## Background

The prevalence of obesity is increasing alarmingly worldwide and is a major health problem as a main leading cause of morbidity and mortality [1]. According to the final report of World Health Organization (WHO) in 2008 more than 1.4 billion of adults were overweight and over 200 million men and approximately 300 million women were obese [2]. The increasing prevalence of overweight and obesity has been defined as a global epidemic [3], which consumption of high fat diet (HFD) is one of the main reasons for this break-out [4, 5]. Experimental studies indicated a linear relationship between the amount of fat intake and weight gain in humans and animal models [6]. In conjunction with its increasing prevalenece, obesity is associated with numerous health-related comorbidities including diabetes, insulin resistance, cardiovascular events and some types of cancers [5]. Among the pathologicaly responsible factors, oxidative stress, defined as an imbalance between tissue free radicals, reactive oxygen species (ROS) and antioxidants, is involved in the etiology of obesity-related pathologic conditions [1, 7]. Obesity, reduces antioxidant enzymes activity includes catalase, glutathione peroxidase (GPx) and glutathione reductase (GR) [1]. High fat diet, is a potent inducer of oxidative stress by altering oxygen metabolism. The fatty deposits as a consequence of high fat diet are vulnerable to suffering oxygen reactions; if the production of these ROS exceeds antioxidant system capacity of the cells, the lipid peroxidation occurs and the lipid peroxidation directly contributes in developing atherosclerosis [8]. The incidence of atherosclerosis or other comorbidities of cardiovascular system is due to oxidative- stress induced endothelial damage and vascular hypertrophy, platelet adhesions and atherosclerotic plaques formation [9]. Moroever, it has been suggested that obesity-induced dyslipidemia leads to tissue damage via oxidative modifications of lipids, protein glycation and glucose auto-oxidation and production of lipid peroxidation metabolits and the dyslipidemia predicts the CVD severity in obese patients [10–12]. Because of the high prevalnec of cardiovascualr disease due to obesity it is important to develop interventional and therapeutic strategies conquering the cardiovascular disaese morbidity in obese individuals [13]. From clinical point of view, the dietary interventions are useful in moderating the inflammatory biomarkers of cardiovascular disease; in the study by Neale et al. [14], the role of healthy dietary patterns in CRP reduction as a main classic cardiovascular risk factor has been indicated. Other studies also reported similar results [15]. However, it will be worth to investigate that which kind of dietary interventions will possibly reduce the markers of oxidative damage and inflammation in patients with cardiac events.

Tissue damage induced by free radicals is thought to be an important factor in the pathogenesis of obesity and obesity related disorders [16]. Measuring the antioxidant defense system in the cardiac tissue is a potent predictor of cardiovascular injury in metabolic disease. In the numerous models of CVD, including congestive heart failure or coronary artery disease, the tissue antioxidant system fails to work and as a consequence, oxygen derived free radicals are increased four-fold and their concentration in the cardiac tissue predicts the severity of heart failure [17]. Elevation of oxidative stress markers and ROS products in the cardiac tissue, leads to direct oxidative damage of cellular components [18].

Vitamin D as a nutrient contained in natural foods, after intake, requires skin exposure to ultraviolet B (290–315 nm) radiation and a series of sequential biochemical reactions occurring in the liver and kidneys to convert 7-dehydrocholesterol into a bio-functional form of vitamin D, which is called vitamin D3 or calcitriol (1,25-dihydroxycholecalciferol) [19].

Although vitamin D is classically well-known for its role in growth and remodeling of bone, recent studies have identified a much broader spectrum of its activity. It is now well recognized that vitamin D, and particularly its active form calcitriol, is an important hormone playing a crucial role in human homeostasis [20]. In 1993, Wiseman first demonstrated that vitamin D is a potent antioxidant vitamin, preventing iron-dependent lipid peroxidation in the cell membrane and acting similar to the cancer drug Tamoxifen [21]. Since then, many studies have conducted to better identify the therapeutic antioxidant roles of vitamin D [22]. It has been shown that vitamin D deficinecy is a potential contributor of cardiovascular deaths. In the LURIC study, as a follow-up of the 3258 participants, patients with severe and moderate vitamin D deficiency were 1.8 to 2.5 times more likely to death from cardiovascular disease compared with patients with normal vitamin D concentrations [23]. In a vitamin D deficient animal model induced by vitamin D deficient diet, vitamin D deficiency increased arterial blood pressure and promoted vascular oxidative stress in rats [24] and was associated with promotion of atrial fibrillation in post-operative patients undergoing coronary artery bypass graft (CABG) surgery [25]. However, considering our review of literature, the role of vitamin D – therapy in health of CVD is a neglected factor.

Moreover, according to above mentioned introduction, it seems that evaluating the role of vitamin D on improving the oxidative damage and inflammation in cardiac tissue has two major benefits; firstly, the results, possibly, will add an important information regarding vitamin D as a factor protecting from oxidative stress and inflammation in cardiac tissue and cardiovascular system that would be easy to add this nutrient in daily clinical practice, and secondly, because of the direct association of markers of oxidative stress and inflammatory parameters with cardiovascular events in human, the results of the current study, could be applicable for using the markers of oxidative stress and inflammation as valuable diagnostic markers in cardiovascular events especially because numerous recent studies reached several debate about using diffused inflammatory markers such as CRP in clinical working; several reports are available showing little or no improvement in the prediction of risk of cardiac events with the addition of CRP to conventional risk factors of cardiovascular disease [26, 27]. Therefore in the current experimental model, we aimed to evaluate the role of vitamin D administration in the cardiac tissue oxidative stress and tumor necrosis factor (TNF) - α concentrations in high-fat diet induced obese rats.

## Methods

The design of study has been mentioned in our previous report [28–30]. Therefore, the characteristics of animals and procedures are reported here briefly.

### Animals, diets and experimental procedures

Forty male Wistar rats weighted 200–220 g were purchased from the Pasteur institute animal care center (Karaj, Iran). The animals were housed five in each cage under standard conditions (light on from 07:00 AM to 07:00 PM and constant temperature of 25 ± 2 °C) with ad libitum access to food and water. Animal experiments were conducted in conformity with the National Institutes of Health ethical guidelines for the care and use of laboratory animals (NIH; Publication No. 85–23, revised 1985) and approved by the veterinary ethics committee of the Tabriz university of medical sciences (Registration number: TBZMED.REC.1395.532). After a week of acclimatization and feeding a standard laboratory chow diet, rats were randomly assigned into 2 groups (*n* = 20, each group): either normal diet (ND) or high fat diet (HFD). ND contained 10% fat, 30% protein and 60% carbohydrate and HFD contained 59% fat, 11% protein and 30% carbohydrate [31]. After four months of receiving ND and HFD, groups randomized in to two subgroups including ND, ND + vitamin D, HFD and HFD + vitamin D, which supplemented with vitamin D or Migliol (Sigma Adrich, USA) 500 IU/kg/d by oral gavage alongside with their prior diets for 5 weeks. Moreover, body weight was weekly measured by scale (PAND Industries, px3000, 5 kg ±1 g) while food intake was monitored 3 times a week.

### Preparation of blood and heart samples

After an overnight fasting, the rats were anesthetized with Ketamin (6.6 mg/kg) and Xylazine (0.3 mg/kg) intra peritoneally. Blood samples were obtained from cardiac puncture and centrifuged at 10000 g at 4 °C for 20 min; sera were separated and stored in an ultra-low temperature freezer (Jal Tajhiz Production, Iran) at −80 °C until assay. Finally, after rats were sacrificed by decapitation, their heart samples were removed and its hemisphere was collected and immediately stored at −80 °C until further use.

### ELISA

Before and after vitamin D supplementation, serum measurement was performed to determine initial and terminal vitamin D level by individual enzyme-linked immunosorbent assay kit (ELISA) (Eastbiopharm, Zhejiang, China) according to the manufacturer’s instructions. Heart tissues were homogenized in phosphate buffered saline (PBS) and centrifuged at 10000 g at 4 °C for 20 min, and clear supernatants were collected and the total protein concentration was measured by protein assay kit (Pars Azmun, Tehran, Karaj). TNF-α concentration in the supernatants were determined using ELISA (Hangzhou Eastbiopharm, Zhejiang, China).

#### Measurement of oxidative stress

The cardiac tissue homogenates were used for determination of glutathione peroxidase (GPx), superoxide dismutase (SOD), malondialdehyde (MDA) and catalase by spectrophotometry in accordance with the protocol provided with the assay kits.

#### Glutathione peroxidase and superoxide dismutase assessment

GPx activity was measured according to Paglia and Valentine method [32] using Ransel, Randox kit (United Kingdom). SOD was assayed by a spectrophotometric method based on the inhibition of a superoxide-induced reduced nicotinamide adenine dinucleotide (NADH) oxidation according to Paoletti et al. method [33] by using Ransod, Randox kit (United Kingdom).

#### Malondialdehyde assessment

Malondialdehyde (MDA) levels were measured using the thiobarbituric acid reactive substances (TBARS) method [34].

#### Catalase assessment

The activities of catalase enzyme were measured according to Hugo aebi method [35].

### Statistical analysis

All statistical analyses were performed using SPSS software, version 16. Kolmogorov–Smirnov test was performed for normality of the distributions of variables. Data are expressed as the mean ± SD. The data were analyzed using one-way analysis of variance (ANOVA) followed by post hoc *Tukey’s* tests and paired sample *t*-test for comparisons between multiple groups and two groups. *P* < 0.05 was considered as statistically significant.

## Results

### Changes in food intake and body weight during the study period

**Fig. 1:**
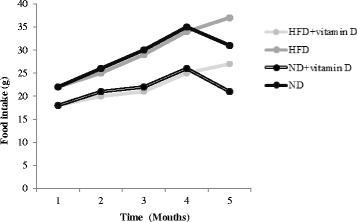
Food intake of studied groups during study period. HFD, high fat diet; ND, normal diet. A reduction in food intake after vitamin D administrations has been occurred in both vitamin D-administered groups (*P* < 0.001)

Changes in food intake of animals during the study period have been shown in Fig. 1. Food intake of animals was gradually increased until the administration of vitamin D and this increase was meaningful in all of study groups. However, after vitamin D administration, in ND and HFD groups, food intake decreased significantly (*P* < 0.001). Accordingly, the baseline body weights were similar among the different groups (Table 1). However, there was a significant difference in body weights of all treated groups at the end of the study (*P* = 0.001). Moreover, *Post-hoc* analysis showed that in the intergroup comparisons of body weight, HFD led to a significant weight gain versus ND group (*P* **=** 0.001).

**Table 1:** Changes in body weight of rats

Groups	1^th^ week	16^th^ week	21^st^ week	^†^ *P* value
ND	218.9 ± 10.83	276.1 ± 26.72	288.9 ± 29.80	*0.001*
ND + Vitamin D	224.6 ± 22.09	278 ± 27.38	255.9 ± 26.90	*0.001*
HFD	218.7 ± 13.27	403.2 ± 4.13	425.4 ± 3.71	*0.001*
HFD + Vitamin D	224.9 ± 13.77	399.8 ± 8.7	380.6 ± 7.80	*0.001*
^‡^ *P* value	0.69	*0.001*	*0.001*	

Data are expressed as means ± SD. Statistical differences between groups were assessed by one-way ANOVA followed by *Tukey’s* test for Post Hoc analysis. Intra group comparisons of body weight were performed by repeated measure analysis. ^†^ *P* value and ^‡^ *P* value indicated intra group and inter group differences, respectively. *P* < 0.05 was considered as statistically significant

### Changes in serum vitamin D concentrations during the study period

Baseline concentrations of vitamin D was not significantly different between groups (*P* = 0.50). As expected, vitamin D administrations led to a marked increase in serum vitamin D concentrations in ND + vitamin D and HFD + vitamin D groups (*P* = 0.001); whereas, serum vitamin D concentrations in HFD and ND groups reduced significantly (Table 2).

**Table 2:** Vitamin D concentrations in study groups

Groups	16^th^ week	21^th^ week	Mean difference	^†^ *P* value
ND	47.50 ± 7.32	36.30 ± 7.74	-11.20 ± 1	*0.001*
ND + Vitamin D	54.87 ± 11.53	119.09 ± 26.01	64.22 ± 7.8	*0.001*
HFD	51.19 ± 11.16	37.70 ± 11.53	-13.49 ± 4.25	*0.01*
HFD + Vitamin D	53.24 ± 14.11	123.70 ± 39.39	70.46 ± 12.85	*0.001*
^‡^ *P* value	0.50	*0.001*		

Data are expressed as means ± SD. Statistical differences between groups were assessed by one-way ANOVA followed by *Tukey’s* test for Post Hoc analysis. Intra group comparisons of serum vitamin D was performed by paired t-test analysis. ^†^ *P* value and ^‡^ *P* value indicated intra group and inter group differences, respectively. *P* < 0.05 was considered as statistically significant

### Changes in serum LDL, HDL, TC and TG concentrations of rats after vitamin D administration

Table 3 presents the effects of vitamin D administration on serum concentrations of low density lipoprotein (LDL), high density lipoprotein (HDL), total cholesterol (TC) and triglyceride (TG) concentrations. Serum LDL concentration after vitamin D administration significantly reduced in ND + vitamin D and HFD + vitamin D compared with ND and HFD groups (*P* < 0.05). Serum TG and TC were also reduced in HFD+ vitamin D group compared with HFD group (*P* < 0.05). No significant change in serum HDL was observed.

**Table 3:** Changes in serum lipids in study groups

Groups	LDL-C (mg/dl)	HDL-C (mg/dl)	TG (mg/dl)	TC (mg/dl)
ND	27.82 ± 5.88	30.08 ± 7.54	41.60 ± 5.01	74.02 ± 3.15
ND + Vitamin D	19.56 ± 5.39	27.64 ± 7.16	50.56 ± 5.02	70.32 ± 3.28
HFD	23.96 ± 5.81	42.50 ± 7.73	54.02 ± 10.51	73.38 ± 7.68
HFD + Vitamin D	19.04 ± 4.63	33.72 ± 9.43	46.14 ± 5.32	65.00 ± 2.47
^†^ *P* value	*0.05*	0.79	*0.043*	*0.034*

Data are expressed as means ± SD. Statistical differences between groups were assessed by one-way ANOVA followed by *Tukey’s* test for Post Hoc analysis. ^†^ *P* value indicated inter group differences. *P* < 0.05 was considered as statistically significant

### Changes in the TNF-α concentration and markers of oxidative stress in the heart tissue

**Fig. 2:**
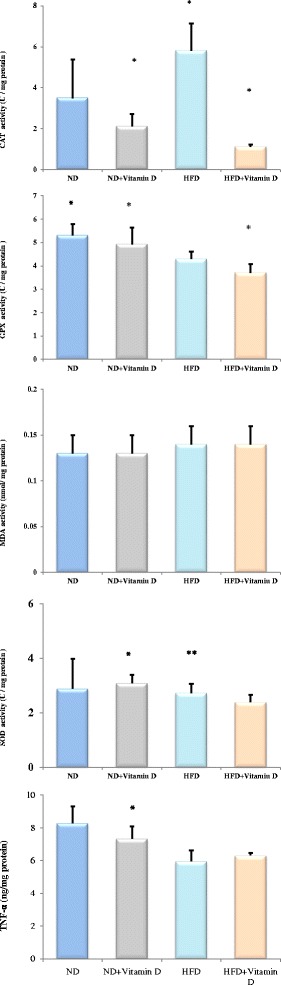
Effects of vitamin D administration on parameters of oxidative stress and TNF-α concentration in heart tissue. * Significant difference between groups (*P* < 0.05)

According to our results (Fig. 2), vitamin D administration, significantly reduced catalase activity in ND + vitamin D and HFD + vitamin D groups (*P* < 0.05). GPx activity in ND and ND + vitamin D group was significantly higher compared with HFD group. Although no difference between ND versus ND+ vitamin D or HFD versus HFD + vitamin D groups in terms of cardiac GPx activity was observed. SOD activity significantly increased in ND + vitamin D group compared with ND and HFD groups. Also, cardiac tissue TNF-α concentration in ND + vitamin D group significantly reduced compared with ND group. No difference in MDA concentration between groups has been observed.

## Discussion

In the present study, we showed that vitamin D administration for 5 weeks has potential cardio-protective effects by modulating biomarkers of inflammation and oxidative stress in cardiac tissue and serum lipids in high-fat diet induced obese rats. According to our results, weight was gradually increased in all groups but HFD group significantly gained more body weight than other groups. Moreover, the present study showed that vitamin D administration protects against cardiac oxidative stress as shown by increased SOD and GPx activity and reduced cardiac tissue inflammation by reducing TNF-α concentration in high fat diet induced obese rats.

The obesity models induced by high fat diet in rats has many common points with human obesity and numerous studies have revealed that antioxidants act as obesity regulators in high fat diet induced obese rats [9]. Obesity is associated with reduced antioxidant defense system in different body organs including heart tissue; in the current research high fat receiving groups had partially increased MDA and reduced antioxidant enzymes activity although these differences were not statistically significant; similarly, several previous reports also confirmed that HFD leads to reduction in serum and cardiac tissue oxidative defense [9, 10, 36]. The antioxidant enzymes like SOD, GPx and catalase scavenge reactive oxygen species and stop their formation; SOD and GPx activities as chief scavengers of anion superoxide (O^2−^) increase after vitamin D administration. In our results, HFD receiving groups had higher baseline catalase activity compared with ND receiving groups and the catalase concentration reduced after vitamin D administration. On the other side, baseline GPx activity was lower among obese rats. Similar to our results, elevated SOD activity was also reported in HFD induced obese rats in the Ansari et al. study [9]. In fact, this finding can be explained by compensatory adaptation of organism to oxidative stress in high fat diet induced obesity. Numerous previous reports have suggested that change in the antioxidant defense system and oxidative stress markers in the body is tissue specific; in other word, as previously suggested by Noyan et al. different body organs might response to oxidative stress selectively by decrease or increase in the concentrations of markers of oxidative stress [37]; increased catalase activity of heart tissue in obese rats, may be an important adaptive response to conditions of increased oxidative stress in the obesity and the biological availability of superoxide anion radicals and hydrogen peroxide in adipose tissue. The increased catalase activity reflects the increased superoxide anion radicals and hydrogen peroxide as catalase decomposes hydrogen peroxide to water and oxygen [38]. In the current work, vitamin D exerted its therapeutic antioxidant roles via decreasing the elevated concentrations of SOD in vitamin D treated rats.

The antioxidant potential of vitamin D is attributed to reduced lipid peroxidation, suppressed nicotinamide adenine dinucleotide phosphate (NADPH) enzyme expression and inhibition of the advanced glycation end products (AGEs) accumulation in the aortic tissue [39, 40]. It has been shown that vitamin D, as an antioxidant molecule, is able to decrease the endothelial impairment after H_2_O_2_ mediated stress, preventing extrinsic caspase cascade activation, switching on MEKs/ERKs/SIRT-1 axis and inhibiting the ROS release [41, 42]. Therefore, vitamin D has potent influence on cardiac endothelial system by affecting oxidative stress system of endothelial cells.

Vitamin D treatment increased SOD activity in ND+ vitamin D group versus ND group; whereas, in HFD + vitamin D group versus HFD group no significant change was observed. This conflict result can be explained by this fact that increased sequestering of the vitamin D occurs in adipose tissue of obese animals and human [43]. As previously reported, for each 1 kg/m^2^ increase in body mass index (BMI) in human, an estimated decrease of 0.74 nmol/L of serum vitamin D has been observed [44] and the vitamin D trapping potential of adipose tissue in obesity limits the functional role of this vitamin by reducing its concentrations in the blood.

Vitamin D exerted its anti-inflammatory actions via reducing cardiac TNF-α concentrations in the current work. Similarly, in the study by Al-Rasheed et al. the effects of vitamin D on TNF-α expressions in rat’s heart had been examined and the results showed that vitamin D protects against cardiac hypertrophy via reducing TNF-α expression by inhibiting NF-кB/p65 signaling pathway [45]. Accordingly, the antagonistic role of vitamin D against TNF-α expression has been confirmed in other studies establishing that vitamin D targets TNF-α pathway to reduce its concentrations in different tissue and organs [46, 47].

Accordingly, vitamin D modified the altered lipid abnormalities and reduced serum LDL, TG and TC concentrations. In fact, dyslipidemia is a very common feature of obesity and a high fat diet is a useful method to induce a model of dyslipidemia in animals or human [48–50]. Therapeutic roles of vitamin D in modification of lipid abnormalities which had been previously confirmed by numerous studies [51, 52] further clarifies the strong cardio-protective potential of this steroid vitamin in treatment of numerous obesity-related disorders.

## Conclusion

The current work revealed that vitamin D ameliorates oxidative stress by increasing the antioxidant enzymes activity and reduces TNF-α concentration in the cardiac tissue of high-fat diet induced obese rats. Therefore the results of the current study further clarify the cardio-protective potential of vitamin D in obesity. Further studies in human models with favorable findings could finally suggest the use of this vitamin in daily clinical practice.

## Abbreviations

AGEs: Advanced glycation end-products
ANOVA: Analysis of variance
CAD: Coronary artery disease
CAT: Catalase
CVD: Cardiovascular disease
GPx: Glutathione peroxidase
HDL: High density lipoprotein cholesterol
LDL: Low density lipoprotein cholesterol
MDA: Malondialdehyde
NADH: Nicotinamide adenine dinucleotide
SOD: Superoxide dismutase
TBARS: Thiobarbituric acid reactive substances
TC: Total cholesterol
TNF-α: Tumor necrosis factor-α
